# Stopping guidelines for an effectiveness trial: what should the protocol specify?

**DOI:** 10.1186/s13063-016-1367-4

**Published:** 2016-05-10

**Authors:** Jon E. Tyson, Claudia Pedroza, Dennis Wallace, Carl D’Angio, Edward F. Bell, Abhik Das

**Affiliations:** Center for Clinical Research and Evidence-Based Medicine, Department of Pediatrics, University of Texas Health Science Center at Houston, 6431 Fannin St, MSB 2.106, Houston, TX 77030 USA; RTI International, Durham, NC 27709 USA; University of Rochester School of Medicine and Dentistry, Rochester, NY 14627 USA; Department of Pediatrics, University of Iowa, Iowa City, IA 52242 USA; Social, Statistical and Environmental Sciences Unit, RTI International, Durham, NC 27709 USA

**Keywords:** Clinical trials, Effectiveness trials, Stopping guidelines, Data monitoring committees, Futility

## Abstract

**Background:**

Despite long-standing problems in decisions to stop clinical trials, stopping guidelines are often vague or unspecified in the trial protocol. Clear, well-conceived guidelines are especially important to assist the data monitoring committees for effectiveness trials.

**Main text:**

To specify better stopping guidelines in the protocol for such trials, the clinical investigators and trial statistician should carefully consider the following kinds of questions:

1. How should the relative importance of the treatment benefits and hazards be assessed?
2. For decisions to stop a trial for benefit:
  - What would be the minimum clinically important difference for the study population?
  - How should the probability that the benefit exceeds that difference be assessed?
  - When should the interim analyses include data from other trials?
  - Would the evidence meet state-of-the-art standards for treatment recommendations and practice guidelines?
3. Should less evidence be required to stop the trial for harm than for benefit?
4. When should conventional stopping guidelines for futility be used for comparative effectiveness trials?

**Conclusion:**

Both clinical and statistical expertise are required to address such challenging questions for effectiveness trials. Their joint consideration by clinical investigators and statisticians is needed to define better stopping guidelines before starting the trial.

## Background

Despite long-standing problems in decisions to stop clinical trials [1–6], stopping guidelines are often vague or unspecified in the trial protocol. The protocol commonly does indicate the number of interim analyses to be performed and the method used to ensure that the type I error does not exceed 0.05 with multiple interim analyses of the primary outcome [7–9]. However, there is usually little effort to clearly indicate when the findings for both the treatment benefits and hazards [6, 10–12] would justify stopping the trial. Moreover, this issue has not been explicitly addressed by the International Conference on Harmonization or such agencies as the Food and Drug Administration [13]. Yet clear, well-conceived guidelines are especially important to assist the data monitoring committees for effectiveness trials [14] intended to determine whether therapies should be used in clinical practice.

## Main text

Definitive stopping rules that cover all circumstances are not possible. However, based partly on our experience in the NICHD Neonatal Research Network, we believe better stopping guidelines can be developed if the following basic kinds of questions are jointly and carefully considered by the clinical investigators and statisticians and addressed in preparing the study protocol:

1. How should the relative importance of the treatment benefits and hazards be assessed?
  The importance (value) of treatment hazards relative to the benefits [15, 16] must be judged in defining stopping guidelines for either benefit or hazards. Although difficult to make, these judgments are implicit in any stopping guideline (as well as any treatment recommendation or practice guideline) [17–20] and are generally based on the views of investigators or clinicians. Ideally these judgments would be based on prior formal assessments of the views of patients (or their surrogates) who have experienced or are most likely to experience these benefits or hazards. This issue is a fertile area for research in increasing meaningful patient involvement in designing clinical trials [21].
2. What evidence should be required to stop the trial for benefit?
  - What would be the clinically important difference (**CID**)—the minimum magnitude of the treatment benefit large enough to offset the treatment harms [16]—for the patient population in the trial?
    The CID depends on the rate and relative importance of treatment benefits and hazards. Clearly and explicitly defined stopping guidelines can specify how the observed rates of treatment benefits and harms can be used in assessing whether the evidence of net benefit or harm is strong enough to justify stopping the trial.
  - How should the probability that the benefit exceeds the CID be assessed?
    While interim analyses are usually performed using only standard frequentist analyses, Bayesian analyses are needed to directly estimate not only the probability of any benefit but also the probability that it exceeds benefit of a specific magnitude such as the CID [22–25]. They may also be used if it is appropriate to incorporate data from prior trials of the same intervention to estimate the updated (posterior) probability of benefit based on the current trial. [See item (c).] In addition, Bayesian analyses are also likely to be more understandable than frequentist analyses to clinicians [23–26]. For these reasons, Bayesian analyses can be recommended for use with conventional frequentist analyses in assessing interim as well as final results [26, 27]. Even so, judgment will still be required in interpreting the analyses and deciding whether the trial should be stopped.
  - When should the planned interim analyses include data from other relevant trials?
    The effect size associated with low *p* values in interim results is likely to be larger than that obtained if the trial is continued to its end [1, 28–31]. At least for trials where early stopping is considered, consideration of data from prior trials and, if appropriate, incorporation into the interim analyses may well be warranted to avoid premature trial termination and erroneous conclusions due to misleading interim data.
  - Would the evidence of benefit meet state-of-the-art standards for treatment recommendations and practice guidelines?
    These standards address many factors beyond statistical significance [19, 20, 32]. At least for comparative effectiveness trials that compare commonly used therapies and are conducted to provide definitive results to guide clinical practice, a strong case can be made to continue the trials if such standards have not been met despite meeting conventional stopping guidelines [1, 2].
3. Should less evidence be required to stop the trial for harm than benefit?
  The prudent answer to this question is likely to be “Yes” based on the ethical maxim of *primum non nocere* and the need to protect patient safety [33, 34] and to focus the limited resources for clinical trials on therapies most likely to be beneficial.
  Yet it is uncertain exactly how much evidence of harm should be required, or in Bayesian terms, what probability of net harm is high enough to stop a trial. For treatment harms as for benefits, interim findings extreme enough to be statistically significant or to have high Bayesian probabilities are likely to be more extreme than those obtained if more patients are studied [6, 28, 35, 36]. Misleading findings are especially likely if the analyses are repeated at frequent intervals. Large numbers of future patients, including some patients who otherwise would have been included in the trial, may then be harmed if one erroneously concludes that a truly beneficial therapy is harmful. Quantitative methods to better address this dilemma are now being explored [37].
4. When should conventional stopping guidelines for futility be used for comparative effectiveness trials?

Conditional power analyses of interim results or Bayesian predictive probabilities [38] help identify when a significant difference between treatment groups is unlikely to be identified if the trial continues to the preplanned sample size. However, such findings would not necessarily indicate that a trial should be stopped. Even in the absence of significant differences, completion of comparative effectiveness trials comparing two widely used therapies may promote greater use of the therapy with the higher likelihood of benefit, particularly if it is also less invasive, hazardous, expensive, or inconvenient. Moreover, important therapeutic questions are rarely answered in a single trial, and early trial termination will reduce the power of later meta-analyses of all relevant trials to identify important treatment effects for all patients, important subgroups, and patients at differing risk [39]. Such analyses are crucial for applying the results of clinical trials to individual patients [15, 16, 39]—a cutting-edge issue important to augmenting the value of clinical trials.

## Conclusions

Although clinical investigators may consider stopping guidelines to be the responsibility of statisticians, both clinical and statistical expertise are required to address such challenging questions. Careful consideration by clinical investigators and statisticians will help to specify better stopping guidelines in the protocol for effectiveness trials, including those that may need especially careful consideration for special populations (e.g., pregnant women or children) or disorders (e.g., cancer). These guidelines can then be reviewed by the data monitoring committee, and any areas of disagreement can be discussed and addressed before starting the trial. As the methods of clinical trials evolve over time, greater input from clinicians and patients will also be needed to promote progressively better informed, better justified, more useful, and more broadly acceptable stopping guidelines.

## Abbreviations

CID: clinically important difference
NICHD: Eunice Kennedy Shriver National Institute of Child Health and Human Development
